# American Dental Association

**Published:** 1896-04

**Authors:** 


### Reports of Society Meetings.

### AMERICAN DENTAL ASSOCIATION.

(Continued from page 169.)

*August 8, 1895.—Third Day.—Evening Session.*

THE meeting was called to order by the vice-president, and the minutes of the previous session read and approved.

Section V. moved that the following resolution be adopted:

*Resolved,* That the American Dental Association condemns the use of secret preparations known as local anæsthetics; that the members thereof pledge themselves to abstain from the use of such nostrums, and all other secret remedies.

Dr. Marshall moved an amendment to include all secret nostrums in which the formula is not printed at the bottom.

Dr. Flagg moved to further amend by leaving out the word "pledge" and to have it merely a resolution of condemnation.

The amendment was accepted by Dr. Marshall.

A report was read by the secretary as to the financial condition of the Dental Protective Association. The committee, consisting of Drs. Smith, Shepard, Noble, and Morgan, find that all funds collected have been accounted for, and all disbursements have been made in the direction of the objects of the Association, and that funds on hand have been securely invested.

The report was adopted.

*Dr. Crouse.*—When we arranged the programme the request was made that one evening should be set apart for the display of stereopticon views, and we arranged for this evening. Since then we find that a paper is to be read to-night; it has been consented to allow the paper to be read, on condition that the discussion would not follow this evening.

Dr. Gillette, of Newport, then read a paper entitled "Cataphoresis for obtunding Sensitive Dentine." He stated that when he read a paper last week before the New Jersey State meeting some gentlemen understood him to claim cataphoresis as a new idea. Dr. Peterson's paper, which he referred to, will give the whole history of cataphoresis, going back to the first experiments in 1858 or 1859. What Dr. Gillette wishes to say concerns cataphoresis in living dentine, and seems to be a plain statement of facts only. Dr. Gillette read some of Dr. Peterson's conclusions in regard to the subject.

The chairman announced as the committee on the furnishing of the museum and library in Washington the following gentlemen: Drs. Donnelly, of Washington; Taft, of Ohio; McKellops, of Missouri; Abbott, of New York; and Morgan, of Tennessee.

In the report of Section IV. Dr. Wilson stated that Dr. Bödecker's researches have been revised and published in book form during the past year. The section has two papers to offer,—one a series of studies on the effect of continued irritation upon the dentine and enamel, by the chairman of the section, Dr. Abbott, and the other by Dr. Vida A. Latham, of Chicago.

Dr. Latham's paper, "A Case of Diffused Non-Suppurative Lymphadentitis," was read by title.

Dr. Abbott's paper was then read, and was followed by the paper of Dr. Cryer, of Philadelphia, entitled "Studies of the Maxillary Bones," which was beautifully illustrated by stereopticon views and lantern slides. The latter paper is from Section VII.

DISCUSSION.

*Dr. Barrett.*—It was my pleasure to examine the specimens of which these are photographs this afternoon. They were a revelation to me, and have modified my views of anatomy as I conceived them before. There are many points which are made clear by the specimens themselves, which are not seen in the photographs. There is probably no practitioner of any experience who has not had cases of diseases in the antrum which to him were incomprehensible. Such a case came into my own practice recently, and I operated three different times, and finally I opened through the external alveolar plate and the alveolar process until I could explore with my finger. I expected to find some foreign substance or some tumor formation. There was absolutely nothing of the kind, and there seemed to be no cause for the amount of discharge that took place. I am satisfied, after seeing Dr. Cryer's specimens, that the probability is that there was a continuous discharge from the frontal sinus through the mass of the ethmoid into the maxillary sinus instead of the nasal sinus. The specimens show so clearly the possibility of a penetration that it seems to me extremely probable that that was the case. The diagnosis of the case which troubled me must have been due to the infundibulum discharging into the maxillary instead of the frontal sinus.

As to the frequent neuralgias which we find in connection with the maxillary nerve, there has been shown by the specimens that not infrequently is it the case that the root of an inferior impacted tooth may penetrate within the inferior maxillary canal and thereby cause all sorts of irritations. There are many other points which have not been brought out to-night which are clearly demonstrated in the specimens themselves. Many of the points that were demonstrated here are of great interest. I wondered this afternoon why I came here. I had not gotten enough for myself to pay me for coming at all,—that is, the papers and discussions which were of interest to others had not been points upon which I was specially interested, but an hour or so with Dr. Cryer paid me more than a hundred times for coming to this meeting. It seems to me, from illustrations of this kind, which incontestably demonstrate some of the aberrations from ordinary normal structure in bone, that those demonstrations will account for a great many of the pathological cases which heretofore have been incomprehensible to us. It has been wonderfully instructive to me, and it must be to any one who attempts to perform even the minor surgical operations of the mouth. They will find the real key to the correct diagnosis of many cases that have heretofore troubled the best pathologists.

*Dr. Marshall.*—I want to express my appreciation of the work of this evening. Especially do I want to thank Dr. Cryer for what he has done in this line. There are so many points that he has brought out that are new and startling that there is not time to speak of all, so I will just refer to one, and that is the location of the opening into the antrum. The later methods of treatment, coming to us from Germany, as to antral diseases, are to open through the nose,—the natural opening. I have made some attempts in that line and succeeded sometimes, but in the majority of cases I have not been able to pass a probe into the natural opening of the antrum through the nose. I have always tried to pass it into the middle meatus, following about an inch and a quarter, working sometimes for an hour (under anæsthetics, of course), and I have failed. I shall not hereafter make any more attempts to work through the natural opening, if the majority of individuals have the opening as shown on the screen to-night.

*Dr. Patterson.*—The extreme pain which we often find resulting from the extraction of impacted, partially erupted wisdom-teeth, lasting for days and sometimes for months, finds ready explanation after this exposition. Their removal requires sometimes a great deal of force. The roots are pressed upon the nerve, which they enter, and consequently are bruised and left in such a condition that pain results for a continued time; nothing but opiates will give the slightest relief. The difficulty in the diagnosis of these pains is by this exposition made simple.

*Dr. Thompson.*—It occurred to me that this could be made interesting by studying the skull from early stages onward. I desire to add my personal thanks and congratulations to Dr. Cryer for his work.

*Dr. Brophy.*—It seems quite likely that Section VII. will not have an opportunity to present anything more during the sessions of this Association than has been presented this evening. As chairman of the section, I feel very proud of the work we have done, and I think we have accomplished a great deal in the presentation of this subject this evening.

In the event of our not being able to bring before the Association the additional matter we have, which amounts to a great deal, we certainly will feel satisfied with what we have done. There is so much to think of in connection with what has been exhibited here by Drs. Cryer and Abbott that it is almost impossible to allude to even a few of the things. I took occasion to interrupt Dr. Cryer when he referred to the inferior dental canal, and want to say that I do not quite agree with the gentleman in changing the name of the passage to a tube. It has so long been known as the inferior dental canal that I am of the opinion it would be better to allow it to retain that name than to suggest a new one. I called attention to the fact that we may have excementosis, and thereby produce the neuralgia which we have in those cases, and which we cannot easily diagnosticate. I believe this is true in the case of children passing through the period of eruption of the teeth, and while it is generally held that the eruption of the teeth is purely a physiological process, there are times when that process is interfered with, and we have a purely pathological condition established from this cause alone. If absorption does not go on equally with the process of development of the root, we have exerted on the inferior dental canal a pressure which will cause the general disturbances which are often found. The relation of the frontal sinus to the antrum opens up a new field for investigation. While it has been known that these relations sometimes exist, I do not think it has ever been so conclusively shown as it has been this evening.

I wish to thank Dr. Cryer for his work in this direction, on my own behalf, and I may also venture to say on behalf of my colleagues engaged in educational work. I would like to know if it is not possible for us to secure slides such as these for the purpose of spreading this information among the students of the various colleges of our country. I do not know that it can be done, but I presume it could. It would greatly facilitate the work of teaching the anatomy of these parts to the students. I think the demonstrations and illustrations this evening will show the value of the careful study of anatomy in our dental colleges.

*Dr. Stellwagen* (of Philadelphia).—I have long desired such a series of sections as we have had exhibited this evening. I have long felt convinced from the examination of a number of these specimens of the mouth, and particularly the superior and inferior maxilla, that this condition which we see here so distinctly illustrated existed,—that the antrum itself was not a simple cavity, but it seemed to be a compound cavity. I found, many years ago, in attempting to treat several sinuses, that it was almost impossible to satisfactorily wash out an antrum with a single opening, unless that opening were very large. Therefore years ago I taught, and I believe I have had such almost universal success that there has been no objection made to it, that in almost every case where there is antral trouble we can succeed in half the time, or perhaps less, in the treatment by having two openings into it. The one which I prefer to use for the purpose of injection and for the purpose of washing out should be from the vestibule of the mouth, from the front of the alveolar process, or from the side, and the other in the roof of the mouth. You will find in many cases where the teeth have been the cause of the complication that there does not require to be any bone removed whatever from the palatal process to pass through it into the antrum, or through the floor of the antrum. In a great majority of those cases this floor itself, so far as the osseous structure is concerned, has changed and portions been removed, so that the mere penetration of the cavity with an ordinary lancet is sufficient, generally preferring a lancet somewhat of the sickle-shape or curved. From the front passing through the process is an operation so trifling that I feel as if it is hardly worth dwelling upon here, and yet in my experience I found a wonderful delicacy or timidity on the part of a number of persons with whom I have conversed on the subject about opening through this little thin plate of bone. To-day, with a bur and engine, I do not think anything of passing through there. I was going to say it is easier to cut through than an ordinary visiting-card. The syringing of the cavity is made as simple as it can be when there are two holes, one for the inflow and one for the outflow of the fluid that is used. I was amazed, a few weeks ago, at a case where the death and subsequent formation of something like cystic opening around the root of a right lateral incisor had passed on, and burrowed and worked its way backward until it had become one with the cavity of the antrum. Seeing the condition almost at a glance, I simply took up a lancet, and opened into the floor of the antrum. I remember a few years ago, when I spoke of doing that to a prominent surgeon of our city, he stated if I did that I would never be able to get it closed. I said, "Suppose I use iodine?" He answered that if I put iodine in, I would kill the patient. In about an hour I had the cavity well washed out with iodine, and the patient showed improvement within a few hours. In a few days the improvement was so marked that I considered her practically well. Sometimes I use a saturated solution of iodine, and sometimes put a few drops in water. If you use a very strong saturated tincture of iodine, do not use quite so much; allow it to be diluted by the contents of the cavity, and if you want to rinse out freely, just put enough into water to have a good color. I lay no importance on the matter of strength in these agents.

In washing out the cavity for this patient a few weeks ago, to my utter amazement, after sending her to the sea-shore for three days, as I thought a little tonic treatment would do her good, at the end of that time the whole trouble appeared to have healed up. I did not attempt to even pass a probe in at the roof of the mouth, for it seemed to have entirely healed. I presume there will always be a fistulous opening, but I look upon it as a very excellent safeguard. This, in my mind, is to all these troubles what a safety-valve is to an ordinary engine. It cannot blow up while that opening is there. Some years ago, the editor of one of our prominent journals brought his wife to me, and wanted an examination made of her mouth. I found a simple fistulous opening of an abscess. She had been under treatment for about three months, having it packed daily, and she was in great dread of some serious complication. I looked at it, and said there was nothing the matter but a fistulous opening, which was a good thing. I told her to leave it alone, and she looked at me in astonishment. I told her to come again, and she came to me occasionally, but never had any more trouble from that fistulous opening than I have from about half a dozen that I have in my mouth.

*Dr. Kirk.*—I have been interested especially in one phase of the discussion this evening. One gentleman said it had been wonderful, another that it was a revelation, and I was quite struck with the reception that this demonstration has received. I do not see anything wonderful or remarkable about it. It seems to me the most natural thing in the world that it should be so. Much of our knowledge of the anatomy of these parts has been erroneous. We have accepted the results of observation of the ancient anatomists with reverence, but they seem to be all wrong about it. Here we have a man who has the brain and the clear insight of things to go right to nature for his facts. I call attention to this thing in connection with the remark of Professor Brophy that it would be of great value if duplicates of these photographs be made. The demonstration is an evidence of the great value of going to nature for our facts.

*Dr. McManus* (of Hartford).—I have been very much interested in this, because it carries out an idea which I have had for a long time, and which was forced on me rather more thoroughly at our legislature, when a physician said any medical man could teach a dentist anatomy and physiology. We have evidence to-night of what a practical, educated dentist can do who sets himself about writing up and illustrating any point in dental anatomy. That is why we are all so pleased that we have had to-night a practical, earnest dentist to show us these things.

*Dr. Cryer.*—I am very much pleased with the remarks that were made as to my endeavor to illustrate that at which I have been working. It has been asked if duplicates of these slides could be obtained. I am under obligation to Lee Brothers & Co. in the matter. Some time ago, Professor Litch asked me if I would write a chapter on anatomy for the "System of American Dentistry." I said I would if they would use new cuts made directly from the subject. Lee Brothers & Co. promised to do so, and these slides are from photographs which will be made into half-tones for this work, and also for another work which will shortly be published. Of course, they would wish this work to come out first before these slides are given out. In a few days I will take my first lessons in photography. Sometimes the photographer does not know what is to be photographed, and I shall then be only too happy to supply the slides that have been asked for to any gentleman for educational purposes.

Adjourned.

*April 9, 1895.—Fourth Day.—Morning Session.*

The meeting was called to order by Dr. Watkins, who presided.

The minutes of the previous session were read and approved.

Dr. Ambler offered the following resolution :

*Resolved*, That it is the sense of this Association that the National Association of Dental Faculties can largely control the formation of dental colleges by passing a resolution refusing to admit any college to their Association of which the organizers did not first apply for and obtain permission from the National Association of Dental Faculties to organize such institution.

Resolution carried.

Dr. Crouse reported that it is the judgment of the Executive Committee on the Horace Wells Memorial matter that the same be, and it is hereby, referred to the Memorial Committee; and further recommend that this Association appropriate $250 for the memorial to Horace Wells, to be paid when the work is completed.

Report adopted.

*Dr. Boice.*—As there are no prospects of Section I. being reached, we would like Dr. Custer's paper on the electric oven to be read by title.

*Dr. Ottofy.*—I wish to introduce this resolution in regard to this matter:

WHEREAS, Dr. Custer made public his invention of an oven for the fusing of porcelain, and made public the process in Chicago and at the Ohio State Dental Society ; and

WHEREAS, Dr. Taggart, of Chicago, also claims the originality of this invention ; therefore be it

*Resolved*, That it is the sense of this Association that, inasmuch as both of these gentlemen are the claimants of this invention, the matter shall be referred to a committee of three, to report at the next annual meeting.

*Dr. Crouse.*—For the purpose of getting the work of this Association better in hand and simplifying it, and having the work of the sections more complete, the Executive Committee would offer the following resolutions:

*Resolved*, That at the session of the American Dental Association, to be held in 1896, the general sessions shall be held every morning, and at such other times as may, at the pleasure of the Association, be designated.

*Resolved*, That the sections shall be required to hold separate meetings simultaneously during the day, and that all papers pertaining to the sections shall be read and discussed in the sections.

*Resolved*, That at the general sessions one hour each session shall be devoted to the president's address, and such other papers as embrace the results of original investigations as may be designated by the proper section.

*Resolved*, That at the general sessions reports embracing only a syllabus shall be made of the papers which have been accepted at the individual sections, meaning hereby that the work must be done in the sections.

*Resolved*, That all papers which have been accepted by the several sections shall be published in the transactions of the session, with the discussions thereon.

*Dr. Marshall.*—This will be a radical change in our order of business, but I think it would be a good one. I am in favor of it.

*Dr. Patterson.*—How can so radical an action be taken without a consideration of one year? It seems to me that it should be done in a very full meeting. Efforts have been made in this direction before, but this matter should have more attention than we could give it at a meeting so slimly attended as the one this morning.

*Dr. Crouse.*—I think there is a law requiring that no paper of over twenty minutes' duration should be read except by title. This would be giving the committee on making up the programme some basis.

*Dr. Fillebrown.*—The members of the Association will bear in mind that it is only a rule for the next year, and it leaves it to the Asssociation at that time to remodel it, if it does not work well. It does not change any organic law, but opens the door for the Association to do as it pleases.

Resolution carried.

Dr. Kirk reported for the Committee on State and Local Organizations. He said, in pursuance of the authorization of the society, copies of the list of topics were forwarded to all the State and local societies of the United States, as far as possible. Acceptances were received from twenty-six societies. Reports of the discussions had upon the topics submitted were received from several of the societies. A number of the societies have promised to furnish reports upon the work, who have not done so owing to lack of time. The committee proposes to refer the report already received to the proper sections of this Association, to be incorporated in the section reports for the ensuing year, and for discussion next year. In view of the interest manifested by the societies in this work, the committee is convinced that this plan, looking to a closer affiliation of the many societies of this country, is a most valuable work, which should be continued on the same lines.

Motion made to accept the report, and the committee continued. Carried.

Dr. Ottofy reported on behalf of the Committee on Credentials, and his report was adopted.

Dr. Taft reported on behalf of the Committee on Necrology, in reference to the death of Dr. John J. R. Patrick and Dr. William O. Kulp; also Sir John Tomes, of England. The report was adopted.

*Dr. Fillebrown.*—The Committee on the Union of the American Dental Association and the Southern Dental Association report progress, and ask for further time. The committee found a general sentiment in favor of the union of the two organizations, with the name and constitution amended to meet the requirements of the present and future.

Report adopted.

*Dr. Shepard.*—Yesterday afternoon, a very enthusiastic meeting was held of the members of the Dental Protective Association, and by unanimous consent of all present, it was considered necessary that the matter should be brought before the American Dental Association, and I was instructed to make a motion that a short time be given to Dr. Crouse to present a short statement of the progress of the suits, etc., and that a few minutes be granted to three or four gentlemen to express their views on the subject to the profession, I move that the order of business be suspended to allow the matter of the Dental Protective Association to be taken up.

Carried.

*Dr. Crouse.*—At the meeting of the American Dental Association last year I described at some length the progress of the litigation. At that time the Dental Protective Association was in pursuit of a contract which existed between the International Tooth Crown Company and James E. Low. I could not find it for a long time, and when I did get it, we found that the Crown Company did not have as much title to it as we even thought they had. Low had not assigned it to the company. When the company was through with the use of the patent they could not dispose of it, but it reverted back to James E. Low. Testimony had to be taken in that regard, and we prepared a brief on that subject, which will be presented to the court, as to the prior use and validity of the patent and of the title. The case will be heard before Judge Wheeler, and it will be argued in Brooklyn. When I left the American Dental Association last year, it was on a summons to appear in New York. I went there and met our attorney there, and we found they were not ready to do anything, and I went home to Chicago. About three weeks later we were again summoned, and we telegraphed to New York to see if it could be adjourned; but not receiving an answer, we started for New York, and at Cleveland we received a telegram that the case would be adjourned; but not trusting to that, we decided that our attorney should go on. We went before the court and were informed that the case would not go on until we had been notified. Our case is in good shape, but having a judicial decision before, we do not know what decision the court may make. The judge may say that as this patent had a judicial decision in its favor, he would refer it back to that federal district in which the decision was obtained; but if he does not, we think he will compel the Tooth Crown Company to begin again, putting in James E. Low as a party to the suit. The case will be heard, one way or the other, within two or three months. If the judge allows the argument in regard to the testimony to go on, and decides on that, we believe we will be successful; but he may refer it to the lower court, and that will necessitate another delay, and bring it before the Federal Supreme Court. In case the decision should go against us, we would appeal; and if we are successful, the Tooth Crown Company would surely do it. We have suits pending with the Steadman patent, which is a removable bridge, and with other patents. I would like it understood that we are not through with the litigation. It is of great importance that we increase our membership, and I would like the other gentlemen to speak about it.

Dr. Jack offered the following resolution:

WHEREAS, This Association has recognized the importance of the work of the Dental Protective Association of the United States by having appointed each year a committee to examine the accounts of the said Association; therefore be it

*Resolved*, That the American Dental Association recommend each dental society of this country to appoint one of its members to solicit subscriptions for membership in the Dental Protective Association.

Carried.

*Dr. Abbott.*—I have been considerably interested in this matter, as a matter of honor for the profession at large,—not personally, because I never made a piece of bridge- or crown-work in my life. I do not know much about it, and I do not want to; but I do want to see these patents that are secured stopped; and I am willing to give not only the ten dollars that is required to become a member, but I will increase that sum if necessary. I want the dental profession to be protected against these sharks that are all over the country. The question has been asked, What has been done with this great amount of money that the president of the Dental Protective Association has gotten into his hands? You will be surprised to know that, with all the work he has done, and the amount he has saved the profession,—over six million dollars in the last eight years,—he has spent less than twenty thousand dollars. Nine-tenths of the dentists in the United States to-day have never paid a cent towards this. Soon somebody will publish the names of the men who are not connected with the Association, and those who hold patents will, no doubt, at once bring suits against all those who have neglected to join the Protective Association.

*Dr. McKellops.*—The dental profession does not seem to have any idea of what Dr. Crouse has been doing. The amount of money that has been saved to the profession is truly wonderful. When we go about through the country, and see these dentists and the suits which are brought against men, costing them from ten to one hundred dollars every year for those patents, we ought to do something about it. Every man should go among his professional brethren and into his society, and see that the men send in their ten dollars each. We need the money, and every man in the profession should make it his duty to put his shoulder to the wheel. I am willing to contribute, for we must have the money, and every man in the Association should think over this thing and take an interest in it. I have labored hard, and have gotten a great many names for the Association. I hope we will hear no more complaint about money, because we must have it.

*Dr. Shepard.*—At the meeting yesterday, which was informally called, there was great enthusiasm, and every one went away imbued with two or three new thoughts. One was the importance of the work, another that it was just and fair to Dr. Crouse that some one else should take up the work, and it was thought that all the future work of soliciting membership for the Association should be done apart from Dr. Crouse. He has raised all this money by his personal efforts, and now we must help. Dr. Crouse could tell you of a dozen suits that will come up. He will tell you of fifteen suits in Denver that he has fought successfully. There are many other patent combinations that are ready to present their demands for royalty, and are only waiting for an opportunity. We must have the money for the conducting of these suits, and also a large reserve fund which can be drawn upon in the next twenty-five or fifty years, or any time for emergencies. One hundred thousand dollars, safely invested, would make every dentist in the country secure from unjust claims,—because all the claims that are fought are unjust claims.

It is well known in the history of these litigations that the monopolies attack some insignificant, humble, honorable member of the profession in a remote locality, without any money and very little nerve, who has one fear in life, and that is that he will be brought into court as a culprit. He is an honest man, but not a rich one, and they will get their judgment in a little district court. They get a hundred such judgments against a hundred feeble men, and the accumulative facts of those minor judgments so helps them that when larger suits are fought there are the decisions of a hundred different petty judges that tell the higher judge that other men have examined the matter and found the claim just. I could name many of them where the original claim had no foundation in justice, and every man knows this to be true. We must help in this matter individually, in our local societies, among our friends, and swell the membership list until it amounts to certainly ten thousand members. In accordance with a vote passed at the informal meeting yesterday, the committee on the Dental Protective Association were appointed to draft an address to the dentists of the country, independent of the Dental Protective Association itself. I will read you the draft of the circular that was proposed. It is to be signed by thirty or forty prominent members of the profession. We must give that support which was given to Moses of old on the mountain, when his hands became tired, to Dr. Crouse, for his hands have become tired; we must give the support to the tired muscle and the wonderful, active, acute brain, which does not yet seem to lose its fineness and its devotion to the interests of the dental profession.

Dr. Jack's resolution was carried.

The chairman called for the discussion of Dr. Gillette's paper, and there being none, the subject was passed.

*Dr. Hoff.*—I want to refer to formaldehyde. It is a very powerful germicide, and one of the best agents for treating root-canals that I know of as a desiccating agent. I have experimented a little with it as an agent for obtunding sensitive dentine, and in my experiments I have found that it is quite beneficial. It has a strong affinity for water, and on that account its use was suggested to me for obtunding sensitive dentine; but I found that if it were allowed to remain any length of time, there would be considerable irritation, especially if it were near the pulp. You can dilute the formaldehyde with chloroform in the proportion of about twenty per cent. of formaldehyde to eighty per cent. of chloroform, and add to this cocaine in various proportions. I have experimented with many proportions, beginning with five per cent. and going as high as thirty per cent. I do not know that I obtained any good results from the cocaine, except that the chloroform, by diluting formaldehyde, makes it last longer. Great care must be exercised, and it should not be left in the cavity too long. Simply wipe out the cavity with the solution, and then dry it out with the hot-air syringe. The operation can be performed with very satisfactory results in ordinary cases. I have not experimented long enough with it to have any very definite data concerning it, but the principle upon which I used it was the faculty it had of taking up water.

*Dr. Guilford* (of Philadelphia).—I have experimented with this, and I find that, in reference to the pulp, it is dangerous. I have removed a pulp with the use of it, and it came away in a leathery condition; so I think we should be cautious about its use. To replace iodoform I have been using for some time a preparation called loeritin, and I have as good success as I had with iodoform. It is odorless, which is a decided advantage.

*Dr. Jack.*—In respect to formaldehyde, I would say that, when it is used for disinfecting the exposed surface of the pulp, it should never be used in a stronger solution than five per cent. of formaldehyde.

Subject passed.

The president appointed on the committee in regard to the investigation of the Custer and Taggart invention the following gentlemen: Drs. Molyneaux, Holland, and James G. Palmer.

Section I. reported a paper entitled "Some Principles relating to Bridge-Work," by Dr. Henry Burchard; the same was read by title.

Dr. Brophy stated that seventy-two papers have been printed on subjects pertaining to anatomy, pathology, and surgery of the oral cavity. It seems unnecessary to read the titles of these papers and the names of the journals in which they were printed, and it would be well to pass this over to the Publication Committee and have it appear in the printed transactions. This seems to be the best report to make of the work of the section, inasmuch as it enables any one who desires to look up the subjects to find them with very little difficulty by simply referring to the transactions.

The section also has a paper by Dr. Barrett, and one by Dr. Ambler, and one by Dr. Brophy, entitled "A New Method of performing the Operation of Staphylorrhaphy."

Dr. Marshall commended the operation of Dr. Brophy, saying that he had used the method for two or three years, and believes that it is one of the best we have.

Dr. Brophy said he had performed this operation in a large number of cases, and it gave better success than any method with which he was acquainted.

Subject passed.

Dr. Peirce stated that Niagara Falls and Saratoga had been suggested for the next meeting. Dr. Weeks showed a communication from the Twin City Commercial Clubs of St. Paul and Minneapolis. These clubs each have a membership of about one thousand members, representing the younger business and professional element of the two cities; they are both social and business in their aims, and they cordially invite the Association to hold its next meeting at St. Paul and Minneapolis.

Dr. Hoff suggested White Sulphur Springs, Virginia, as a desirable place.

The society proceeded to vote for the place of meeting for 1896, and Saratoga was found to be the choice. Drs. Hoff and Luckey were the tellers.

*Dr. Stockton.*—We have heard with great regret of the serious illness of our beloved president, Dr. Crawford. He said it was the regret of his life that he was unable to preside at this meeting. I remember eight or nine years ago that our then president, Dr. McKellops, was very ill during the time of our meeting. We re-elected him, and it was just the elixir he needed, for he became well again. I think it is the elixir that Dr. Crawford needs, and I move that the secretary cast one vote for Dr. Crawford as president.

Dr. Crouse objected, and the regular balloting was then proceeded with, with the result that Dr. Crawford was elected.

A committee was appointed to immediately inform President Crawford of his re-election, and Drs. Frederichs, Stockton, and Patterson were appointed on such committee.

The other officers elected were, First Vice-President, Dr. McManus; Second Vice-President, Dr. Fillebrown; Recording Secretary, Dr. Cushing; Corresponding Secretary, Dr. Chase; Treasurer, Dr. Morgan.

*Executive Committee.*—Drs. Crouse, Ottofy, and Jackson.

The committee appointed to wait on Dr. Crawford and inform him of his re-election, having returned, reported that they were unable to see him, but saw Mrs. Crawford, who informed them that Dr. Crawford was somewhat better. She expressed her gratification at the re-election, and said that the committee could say that Dr. Crawford would accept the same.

Dr. Fillebrown read a letter from Dr. Crawford in which he expresses his regret at being unable to preside at the meeting, and expressing his thanks to the officers for their efficient work during the past year, and particularly to Dr. Emma Eames Chase, the corresponding secretary.

Dr. Donnelly moved a rising vote of thanks to Dr. Watkins, the first vice-president, for the able and impartial manner in which he has presided over the meeting.

Dr. Fillebrown was called to the chair, to represent the installation of the newly-elected president.

Dr. Watkins thanked the society for the courteous way in which they had treated him while he presided over their meetings.

Drs. Harlan and E. T. Darby were appointed on the Publication Committee, the secretary of the Association being *ex officio* a member of such committee.

The secretary read the final minutes of the session.

Dr. Jackson reported that the Auditing Committee had examined the books of the treasurer and also audited the accounts and found them correct.

Report accepted.

The meeting thereupon adjourned to the first Tuesday of August, 1896.

